# Gut Microbiota: A Contributing Factor to Obesity

**DOI:** 10.3389/fcimb.2016.00095

**Published:** 2016-08-30

**Authors:** Steve M. Harakeh, Imran Khan, Taha Kumosani, Elie Barbour, Saad B. Almasaudi, Suhad M. Bahijri, Sulaiman M. Alfadul, Ghada M. A. Ajabnoor, Esam I. Azhar

**Affiliations:** ^1^Special Infectious Agents Unit, King Fahd Medical Research Center, King Abdulaziz UniversityJeddah, Saudi Arabia; ^2^Department of Biochemistry, Faculty of Science, King Abdulaziz UniversityJeddah, Saudi Arabia; ^3^Experimental Biochemistry Unit, King Fahd Medical Research Center, King Abdulaziz UniversityJeddah, Saudi Arabia; ^4^Department of Animal and Veterinary Sciences, Faculty of Agricultural and Food Sciences, American University of BeirutBeirut, Lebanon; ^5^Department of Biochemistry, Faculty of Science, King Abdulaziz UniversityJeddah, Saudi Arabia; ^6^Biology Department, Faculty of Science, King Abdulaziz UniversityJeddah, Saudi Arabia; ^7^Clinical Biochemistry Department, College of Medicine, Nutrition Unit-King Fahd Medical Research Center, King Abdulaziz UniversityJeddah, Saudi Arabia; ^8^King Abdulaziz City for Science and TechnologyRiyadh, Saudi Arabia; ^9^Department of Medical Laboratory Technology, Faculty of Applied Medical Science, King Abdulaziz UniversityJeddah, Saudi Arabia

**Keywords:** Saudi Arabia, obesity, gut microbiota, food, GM-obesity dilema

## Abstract

Obesity, a global epidemic of the modern era, is a risk factor for cardiovascular diseases (CVD) and diabetes. The pervasiveness of obesity and overweight in both developed as well as developing populations is on the rise and placing a huge burden on health and economic resources. Consequently, research to control this emerging epidemic is of utmost importance. Recently, host interactions with their resident gut microbiota (GM) have been reported to be involved in the pathogenesis of many metabolic diseases, including obesity, diabetes, and CVD. Around 10^14^ microorganisms reside within the lower human intestine and many of these 10^14^ microorganisms have developed mutualistic or commensal associations with the host and actively involved in many physiological processes of the host. However, dysbiosis (altered gut microbial composition) with other predisposing genetic and environmental factors, may contribute to host metabolic disorders resulting in many ailments. Therefore, delineating the role of GM as a contributing factor to obesity is the main objective of this review. Obesity research, as a field is expanding rapidly due to major advances in nutrigenomics, metabolomics, RNA silencing, epigenetics, and other disciplines that may result in the emergence of new technologies and methods to better interpret causal relationships between microbiota and obesity.

## Introduction

Pervasiveness of obesity and the other disorders associated to this, for example, metabolic syndrome, have become a great challenge for health care throughout the world (Kovatcheva-Datchary and Arora, 2013). Obesity is a multifaceted and multi-factorial condition that results from the interaction of one's genotype with environmental exposures.

It was estimated by the World Health Organization that 1.9 billion adult people were overweight, out of which 600 millions were obese in 2014. Futhermore, recent estimates of 2014 indicate that, since 1980, incidence of obesity has more than doubled (WHO, 2015). Globally, obesity is rising as an epidemic and is identified to be an emerging public health threat in the Middle Eastern region (Lobstein et al., 2004). In the United States, obesity and overweight individuals are prone to premature mortality, possibly arising from multiple etiologies (Fat et al., 1998) while in Canada, 57% of adult men and 35% of adult women are overweight or obese (Canning et al., 2004). Recent data reported that around 18.3% of the adult Canadian population could be classified as obese (Twells et al., 2014). Moreover, it was reported in 2007 that about 40% of adults in Tehran were overweight and 23.1% of these individuals were obese (Rezaeian and Salem, 2007). The problem is more severe in most of the European countries. Data collected from European countries claimed that around more than half of the European population were obese or overweight (Eurostat, 2015). Latest national data from the USA as of November 2015, on obesity among adults and youth revealed that around 36% of adults and 17% percent of children and adolescents are obese (Ogden et al., 2014).

Obesity has appeared as an endemic disorder that is prominent in developed countries and is quickly emerging as a major problem in some of the developing countries such as Saudi Arabia.

For the past three decades, Saudi Arabia has experienced considerable economic growth drastically impacting lifestyle of citizens, specifically eating and exercise habits (Darwish et al., 2014) giving rise to major nutritional changes among the Saudis (Musaiger, 2011) leading to a higher prevalence of overweight and obesity to alarming levels (Al-Nuaim et al., 1997). According to the local news agency, around 70% of adult Saudis are obese or overweight (Arab News, 2014).

A study conducted in 2012 indicated that percent body fat among Saudi children and teenagers is rising and thus resulting in an increase in obesity (Al-Hazzaa et al., 2012). The estimates show that Saudi children and teenagers are 26.6 and 10.6% overweight or obese, respectively (El Mouzan et al., 2010). Saudi adolescents between the ages from 15 to 19 years were reported to suffer from sleep deprivation and thus are exposed to high risk of overweight and obesity (Al-Hazzaa et al., 2012).

Recently, the resident microbial communities, known as Gut Microbiota (GM), has been suggested to be a major driving force in the pathogenesis of metabolic disease and obesity, in particular (Kovatcheva-Datchary and Arora, 2013; Khan et al., 2014; Nguyen et al., 2015). Besides genetic, environmental, and the immune system related factors, GM is one of the contributing factor in developing obesity (Giovanni et al., 2010). Thus, scientific interest has been directed toward understanding the contributions of GM in obesity and metabolic disease. The GM exerts their role through several integrated pathways including the host immune system, responses to the environment including diet and their genome in addition to several factors (Ferreira et al., 2011; Khan et al., 2014).

Around 10^14^ microorganisms colonize various parts of the human body and human body is estimated to be around 90% bacterial cells of all cells (Khan et al., 2014). Generally, they colonize exterior and interior mucosal surfaces of the human body. Microbes colonizes skin, gastrointestinal, genitourinary, and upper respiratory tracts as well as other sites on the human body. They are found in high concentrations in the lower gastrointestinal tract (Frank et al., 2007; Hattori and Taylor, 2009). The GM are primarily bacterial though fungal, protozoan, and archaeal species have been isolated too (Khan et al., 2014). Furthermore, the collective metagenome content is 150-fold larger on a gene basis than that of the human genome. Bacteria constitute 99% of the total GM, in which 90% fall under two main phyla the Firmicutes and Bacteroidetes. However, a small population of bacteria belongs to the phyla Proteobacteria, Actinobacteria, Verrucomicrobia, and Fusobacteria (Khan et al., 2014).

Humans have coevolved and thus developed several symbiotic associations with their GM that accounts for the high concentrations of GM. The GM association with their hosts could be commensal (negligible net effect on host physiology) leading to enhancing the overall ability of the body to be a better fit for survival. It has been reported that GM plays an important role in enhancing the digestion and absorption of otherwise indigestible nutrients as well as energy turnover (Dethlefsen et al., 2006; Round and Mazmanian, 2009; Foster and Neufeld, 2013). GM diversity and composition are profoundly influenced by host diet, lifestyle, and environmental factors (Maslowski and Mackay, 2011; Graf et al., 2015). Advanced analytical platforms such as metagenomics and metabolomics, revealed that GM help host in harvesting energy and lead to increasing adiposity (Dumas et al., 2006; Martin et al., 2008; Turnbaugh et al., 2008; Turnbaugh and Gordon, 2009). Accordingly, a 20% increase in Firmicutes and a corresponding decrease in Bacteroidetes were found to be associated with the increase in energy intake, thus inducing obesity (Rautava et al., 2012). GM population surveys primarily are basedon clinical data, as well as data obtained from gnotobiotic mice and epidemiological studies. This includes studies conducted on various human populations and risk groups such as formula vs. breastfed infants and Cesarean- vs. vaginally delivered infants (Koenig et al., 2011; Maynard et al., 2012). However, emerging research indicates that modern dietary, hygienic, and medicinal practices strongly affect the variations in GM composition. GM composition is involved with a number of chronic diseases and ailments including obesity among populations in the developed countries (Backhed et al., 2004; Turnbaugh et al., 2006), diabetes (Ehehalt et al., 2010), inflammatory and immune disorders (Penders et al., 2007), and cancer (Zhang et al., 2012; Khan et al., 2014). This review examines the contributing role of GM in the development of obesity. Various factors are discussed that contribute to the development of obesity.

## Diet effects on GM composition

Despite multiple paths and physiological factors, obesity is tightly linked to diet, and lifestyle that could promote several other metabolic disorders if not properly managed (Kovatcheva-Datchary and Arora, 2013; Boursier and Diehl, 2015). It is now a fact that GM is playing important role in harvesting energy efficiently from the diet (Khan et al., 2014; Graf et al., 2015). The GM participates within various biochemical pathways to assist in digestion and metabolism of food. Interestingly, the GM of obese individuals exhibits aberrant carbohydrate and lipids metabolism (Rautava et al., 2012; Caesar et al., 2016). Recent studies showed that the consumption of diet emulsifiers have been associated with changes in the GM composition (Chassaing et al., 2015). Carbohydrates are a vital source of dietary energy. However, humans are not capable of digesting all linkages inherent to oligo- or polysaccharides. Non-digestible carbohydrates include plant-derived fibers such as xylans, cellulose, inulin, and resistant starch. GM degrades these carbohydrates for harvesting energy and providing the host with a variety of metabolites such as short-chain fatty acids (SCFAs) propionate, acetate, and butyrate (Tremaroli and Bäckhed, 2012). These SCFAs affect glucose, cholesterol, and lipid metabolism in different body tissues (den Besten et al., 2013). Polysaccharide fermentation by GM impacts host adiposity through modulating energy derived from dietary substrates (Tremaroli and Bäckhed, 2012).

The type of food intake by the human host influences the GM composition and diversity (Carmody et al., 2015; Gérard, 2016). For instance, the western diet (high fat) results in a reduction of Bacteroidetes and an increase in Firmicutes, especially Mollicutes (DiBaise et al., 2012). The latter is associated with an enrichment genes involved in carbohydrate catabolism (Turnbaugh et al., 2008; Bested et al., 2013). Accordingly, obese individuals are known to have a microbiota rich in Firmicutes and lower in Bacteroidetes as compared to the GM of lean individuals (Scott et al., 2015). Similarly, Prevotellaceae, a hydrogen-producing bacteria, and archaeal species were abundant in obese individuals. It was demonstrated that the transfer of hydrogen between archaeal and bacterial species might enhance energy harvest efficiency within the gut (DiBaise et al., 2012; Doré and Blottière, 2015). Interestingly, high-fat diets modulate the microbiome composition to increase circulatory lipopolysaccharides coinciding with general inflammation (Cani and Van Hul, 2015). General dysbiosis within the gut is associated with a high level of plasma endotoxin and inflammation that eventually promotes metabolic disorder (Zhang et al., 2010, 2013). Zhang et al. (2013) showed that one of the endotoxins producing bacteria (i.e., Enterobacter), when inoculated into germ-free mice resulted in the induction of obesity and insulin resistance (Zhang et al., 2010). Moreover, the host genetics, diet, and different environmental factors influence the etiology of liver disorders. As such, the GM has been recognized as being important in greatly influencing the host metabolism. GM may have a double-edged sword and may exert both beneficial and deleterious extra-intestinal influence to the host.

Furthermore, GM actively participate in bile acid (BA) activation, metabolism, and regulation (Sayin et al., 2013). BA is secreated from liver to digestive system and help in lipid absorption (Inagaki et al., 2005; Kuribayashi et al., 2012). Abnormal BA level has been linked with various metabolic diseases including obesity (Kuribayashi et al., 2012). Gut bacteria, especially *Clostridium scindens, Clostridium sordellii*, and *Bacillus fragilis* have a proven role in the biotransformation of BA (Miyata et al., 2009). Any aberration in GM composition can modulate BA level and can accordingly manipulate obesity (Ridlon et al., 2014). However, theantimicrobial activity of conjugated-BAs is suppressed during deconjugation through the bile salt hydrolase enzyme, which is excreated by the GM, especially bacteria from the phyla *Firmicutes, Bacteroidetes*, and *Actinobacteria*.

## GM modulate energy intake

Obesity is a consequence of a prolonged imbalance between energy intake and expenditure caused by a complexinterplay between genetic susceptibility and nutritional, physiological, social, and environmental factors (Brahe et al., 2016). In addition, it has been estimated that 40% of the gut microbial gene pool is shared among individuals (Qin et al., 2010), and there seems to exist a core microbiome, which is a set of microbial genes shared among the vast majority of healthy individuals that enables conservation of several important functional pathways including those involved in energy metabolism (Brahe et al., 2016). The connection between GM and energy homeostasis and inflammation and its role in the pathogenesis of obesity-related disorders are becoming increasingly recognized.

Mammalian evolution includes innovation of mechanisms to store energy in the form of adipose tissue for when needed and providing physical protection during exercise (Chaput et al., 2010). Maintaining energy homeostasis requires strict regulation of energy intake and the amount consumed. Obesity has increased in developed countries, in part, due to lifestyle changes and the availability of energy high caloric foods. A significant change in GM composition has been reported to be associated with obesity. In addition, germ-free mice do not develop diet-induced obesity. Such a finding indicates that the effect of GM on the host metabolism and obesity induction is achieved by more efficient harvest of energy yield from consumed food and modulating dietary or the host-derived compounds that alter the host's metabolic pathways (Tremaroli and Bäckhed, 2012; Graf et al., 2015).

No significant increase in weight was observed when germ-free mice were fed a high-fat diet, intestinal SCFAs were reduced, and calories were lost through urine and fecal elimination (Brun et al., 2007; DiBaise et al., 2012).

It has been clearly demonstrated in animal models that GM play a role in inducing obesity through a more efficient way for better energy yield from consumed foods and by modulating dietary or the host-derived compounds that alter host metabolic pathway (Tremaroli and Bäckhed, 2012; Table 1). The same results were observed after a patient had a Roux-en-Y gastric bypass operation, where there was an increase in the presence of Prevotella and Bacteroides which correlated in a negative manner with energy intake and adiposity (Kovatcheva-Datchary and Arora, 2013). Accordingly, The increased ratio of *Bacteroidetes* to Firmicutes was linked with diminished body mass (De Filippo et al., 2010; Kovatcheva-Datchary and Arora, 2013). There are several Firmicutes genera/species typically isolated or observed in the gut. This includes various genera associated with Clostridia class (e.g., *Ruminococcus*) that may secrete butyrate. In general, the primary Bacteroidetes genera associated with the gut are *Bacteroides* spp. and *Prevotella*. The same results were observed when a patient received a gastric bypass operation with Prevotella and Bacteroides were augmented with reducing adiposity (Yatsunenko et al., 2012; Kovatcheva-Datchary and Arora, 2013). There is, however, disagreement in the scientific literature (Schwiertz et al., 2010). The role of GM composition in relation to obesity is not clear. This is because there are different etiological factors that are behind obesity and can be linked to different microbes (Tremaroli and Bäckhed, 2012).

**Table 1:** **List of some bacterial taxa and their association with obesity**.

**Bacterial taxa**	**Association with obesity**	**References**
*Firmicutes*	Associated with genes involved in carbohydrate catabolism and rich in obese individuals. It was dominated the GM of European children. After gastric bypass surgery *Firmicutes* level got decreased.	Turnbaugh et al., 2009; DiBaise et al., 2012; Bested et al., 2013
*Enterobacter*	An endotoxin producing bacteria which inoculated into germ-free mice resulted in the induction of obesity and insulin resistance.	Zhang et al., 2015
*Bacteroidetes* (mainly include *Bacteroides* spp. and *Prevotella*)	Is linked with diminished body mass. It dominated the microbiota of children from rural African villages. It got enriched in the GM of mice consuming a high fiberdiet as they are efficient utilizers of plant polysaccharides.	De Filippo et al., 2010; Kovatcheva-Datchary and Arora, 2013
*Prevotella* and *Bacteroides*	Augmented with reduce adiposity in patients who received gastric bypass operation.	Yatsunenko et al., 2012; Kovatcheva-Datchary and Arora, 2013
*Lactobacillusrhamnosus* PL60	Conjugated linoleic acid (CLA-probiotic bacteria) and maintains the body weight of mice fed a high fat diet.	Kovatcheva-Datchary and Arora, 2013
*Escherichia coli*	Linked to the absence of obesity.	Million et al., 2013a
*Lactobacillus reuteri*	Positively linked with obesity and after using ready-to-use therapeutic food containing this bacterium the weight gain was observed in children suffering from Kwashiorkor disease.	Million et al., 2013b
*Faecalibateriumprausnitzii*	Negatively associated with inflammatory markers that can alleviate obesity.	Tremaroli and Bäckhed, 2012
*Bacteroides thetaiotaomicron*	Capable of degrading dietary polysaccharides and fermenting fructans to acetate that further promotes adipogenesis in mice.	Conterno et al., 2011
*Prevotellaceae*	Hydrogen-producing bacteria, and archaeal species were abundant in obese individuals.	DiBaise et al., 2012; Doré and Blottière, 2015
	It was demonstrated that the transfer of hydrogen between archaeal and bacterial species may enhance energy harvest efficiency within the gut.	
*Staphylococcus aureus*	Its presence in pregnant woman may increase the risk of obesity in their progeny due to vertical transfer of bacterial populations during birth or rearing.	Vaughan et al., 2002; Kalliomäki et al., 2008
*Bifidobacteria*	Dietary oligosaccharides derived from plant and milk stimulates the growth of this bacteria.	LoCascio et al., 2007; Meyer and Stasse-Wolthuis, 2009
*Bifidobacterium* spp. and *Bacteroidetes*	Decrease in obese pregnant women.	Santacruz et al., 2010
*Prevotellaceae*	Increase in obese pregnant women.	Santacruz et al., 2010
*Firmicutes/Bacteroidetes*	Ratio was high in obese individuals compared to healthy subject.	Santacruz et al., 2010
*Methanobrevibacter smithii*	Associated with weight gain in children.	Mbakwa et al., 2015
*Bifidobacteriumanimalis* and *Lactobacillus reuteri*	Former was linked with normal weight gain and was found in low concentration in obese individuals but the latter was reportedly increased in obese patients.	Million et al., 2012
*Weissellakoreensis*	Bacterial specie that is abundantly found in Kimchi and showed anti-obese effect via production of ornithine.	Kang et al., 2013
*Lactobacillus gasseri* SBT2055 (LG2055)	The administration of this bacterium to HFD-induced obese male mice prevents the gain of body weight by suppressing the expression of the CC chemokine ligand 2 (Ccl2) gene in the adipose tissue of obese mice.	Ukibe et al., 2015
*Staphylococcus aureus*	High statistical association between body mass index (BMI) and *Staphylococcus aureusws* observed in female prisoners.	Befus et al., 2015
*Lactobacillus casei* CRL 431	The administration of this bacterium to diet-induced obese mouse led to lowering of weight gain.	Nunez et al., 2014
*Lactobacillus paracasei* CNCM I-4270 and *Lactobacillus rhamnosus* I-3690	Attenuate weight gain in high fat diet (HFD)-induced obese mice.	Wang et al., 2015
*Lactobacillus rhamnosus* CGMCC1.3724	Supplementation of this bacterium to obese women helps achieve sustainable weight loss.	Sanchez et al., 2014
*Lactobacillus plantarum* LG42	Showed anti-obese effect when administered to high fat diet (HFD)-induced obese mice.	Park et al., 2013
*Bifidobacterium breve* B-3	Lowers body weight in diet induced obese mouse.	Kondo et al., 2013
*Lactobacillus curvatus* HY7601 and *Lactobacilllus plantarum* KY1032	Reduced body weight of diet-induced obese mice.	Park et al., 2013
*Lactobacillus gasseri* BNR17	Showed anti-obese effect.	Kang et al., 2013
*Akkermansia muciniphila*	Showed inverse relation with obesity in human and diet-induced obese mice.	Roopchand et al., 2015; Schneeberger et al., 2015

Conjugated linoleic acid (CLA) is a derivative of linoleic acid that is naturally found in dairy products and has been involved in increasing the metabolic rate in mice (West et al., 1998; Kovatcheva-Datchary and Arora, 2013). The CLA-producing probiotic bacteria (*Lactobacillus rhamnosus* PL60) maintain the body weight of mice fed a high-fat diet. The same diet had resulted in induction of obesity in mice that were not colonized with either *L. rhamnosus* or *Lactobacillus plantarum* (Kovatcheva-Datchary and Arora, 2013). Similarly, *Escherichia coli* were linked to the absence of obesity (Million et al., 2013b). In contrast, there are bacteria that have a positive linkage to obesity, such as *Lactobacillus reuteri* and were linked to weight gain in children affected with Kwashiorkor using ready-to-use therapeutic food (Million et al., 2013a). Moreover, certain strains like *Faecalibaterium prausnitzii* which are less abundant in obese and diabetic patients may modulate obesity through different mechanisms which are negatively correlated with inflammatory markers proving that that *F. prausnitzii* may lead to the modulation of obesity (Tremaroli and Bäckhed, 2012).

GM has the potential to synthesize a vast amount of enzymes like glycoside which hydrolyses the breakdown of complex plant polysaccharides to monosaccharides. This is in addition to the production of ligands made of short-chain fatty acids like acetate, propionate, and butyrate. When these ligands for two G-proteins-coupled receptors Gpr41 and Gpr43, of gut enteroendocrine cells bind, they result in the stimulation of the gut peptide PYY which inhibits gut motility and slows intestinal transit and it allow the better absorption of nutrients and may be used as a treatment for obesity (Batterham et al., 2003).

## Obesity can be regulated by modulating GM

The hypothesis that obesity could be controlled by guiding the composition and function of GM holds much potential for therapeutic interventions (Burcelin et al., 2015; Wang et al., 2015). For example, *Bacteroides thetaiotaomicron* is capable of degrading dietary polysaccharides and fermenting fructans to acetate that promotes adipogenesis in mice (Conterno et al., 2011). In addition, the co-colonization of *B. thetaiotaomicron* with other fermentable strains such as *Methanobrevibacter smithii* can potentiate the process of adipose tissue build-up and thus potentiates obesity (DiBaise et al., 2012).

In order to control obesity in relation to the GM composition, certain ecological and functional parameters remain to be fully elucidated (Cani and Van Hul, 2015; Brahe et al., 2016). Investigations into the ecological role of specific and individual microbiota populations will likely yield targets for probiotic interventions in communities that are incorrectly assembled (Carding et al., 2015). There are several probiotic strategies that have been developed containing *Lactobacillus acidophilus* tailored toward reducing the serum cholesterol levels in humans (Parvez et al., 2006; Cani and Van Hul, 2015). Interestingly, in a study to characterize possible determinants for childhood obesity, it was found that the common skin bacterium *Staphylococcus aureus* in feces was a marker for obesity risk during development (DiBaise et al., 2012). In a similar study of pregnant women, overweight individuals harbored higher concentrations of *S. aureus*. This may increase the risk of obesity in their progeny due to a vertical transfer of bacterial populations during birth or rearing (Vaughan et al., 2002; Kalliomäki et al., 2008).

Based on animal studies, it was reported that the G-protein-coupled receptor (GPR) deficient mice were abundantly colonized by two commensals bacteria i.e., *B. thetaiotaomicron* and *M. smithii*. Those mice were significantly leaner than their wild-type counterpart littermates. This is possibly because Gpr deficient mice had a lower expression of peptide tyrosine tyrosine (PYY), fast intestinal rate, and reduced energy intake from the consumed food. There was an increase in the glucagon-like peptide (GLP)-1 as a response to GM fermentation of prebiotics, which promoted L-cell differentiation in the rat's colon. The treatment of ob/ob mice with prebiotic crbohydrates has resulted in the alteration of the GM composition and an increase in the circulating GLP-1 and GLP-2. The GLP-1 was important in mediating the prebiotic action and prevented its effects on body weight gain, glucose metabolism, and inflammatory pathway activation. Studies showed that GLP-2, a 33-amino acid peptide, may cause the GM to modulate the gut barrier and endotoxinemia and is known for its intestinotrophic properties (Samuel et al., 2008; De Silva and Bloom, 2012).

The presence of *Akkermansia mucin* I Phila is associated with a more efficient glucose metabolism and leanness in mice. However, this has not yet been proven in humans. The new findings indicate that the higher abundance of *A. mucin* I Phila results in healthier metabolic status in overweight/obese humans and is associated with improved glucose homeostasis, blood lipids, and body composition after calorie restriction. The current data need to be investigated further to allow for a more conclusive link to ascertain the therapeutic applicability of *A. muciniphila* in the treatment of insulin resistance. In addition to the possible use of *A. muciniphila* as a diagnostic or prognostic tool to predict the potential success of dietary interventions (Dao et al., 2016). Obesity is likely to be related to: (a) an increase in *Firmicutes* phylum, (b) a reduced abundance of *Bacteroidetes*, (c) a higher level of *Actinobacteria* phylum, (d) a Lower proportion of *Verrucomicrobia*, and (e) a reduction in the abundance of *Faecalibacterium prausnitzii* spec. (Chakraborti, 2015).

## Relationship between GM and obesity

There are several well-established risk factors for obesity including genetics, lifestyle, and dietary habits. However, recently, and due to new advances in nucleic acid sequencing technology, there has been an emerging field to examine GM's role in obesity. Fecal transplantation studies conducted on mice, humans, and human-to-mouse have led to several molecular links between GM composition and obesity (Conterno et al., 2011). It has been reported that a healthy woman (BMI 26) who had contracted a reoccurring *Clostridium difficile* infection undergone fecal transplant from her overweight yet healthy daughter became obese (BMI 34.5) 36 months after receiving the fecal transplant. The mother failed to lose weight after following a “medically supervised liquid protein diet and exercise program” (Brandt, 2012; Alang and Kelly, 2015).

Inter-individual variation in microbiome composition and methodological tools has led to some difficulties in reconciling large taxonomic surveys of healthy or obese associated microbiota. This includes impactful studies conducted by the Human Microbiome Project and the Metagenomics of the Human Intestinal Tract (MetaHIT). Thus, a scientific consensus on what constitutes a conserved obese enterotype is not fully realized.

In obese patients, a bimodal distribution of microbial gene richness has been noted. Obese individuals are stratified as either having high Gene Count (HGC) or Low Gene Count (LGC). A high prevalence of presumed anti-inflammatory species of *F. prausnitzii* was noted in 32 HGC individuals with higher production potential of like butyrate as well as other organic acids. A different pattern was seen in LGC individuals who demonstrated a higher relative abundance of potentially proinflamatory *Bacteroides* spp. and genes associated with oxidative stress response. A significant association of biochemical obesity-associated variables, such as insulin resistance, was observed with gene counts. However, weight and BMI did not show such an association. Such a phenomenon underscores the inadequacy of using BMI as an indicator of “Obesity and its Associated Metabolic Disorders” (OAMD). Later it was shown that in a diet used for the induction of weight-loss significantly resulted in higher gene richness in individuals with LGC associated with improved metabolic status. Although gene richness was not fully restored, the data further support the reported link between GM structure and the long-term dietary habits. Based on that, it was concluded that diet can play a crucial role in the permanent adjustment of the microbiota (Marchesi et al., 2016).

It was published that GM-promoted storage of circulating triglycerides into adipocytes. Such an activity was caused by suppressing the intestinal secretions of an inhibitor of adipose tissue lipoprotein lipase FIAF, which is angiopoietin-like protein. Consistently, in FIAF-deficient knockout (KO) mice, only a 10% increase in total body fat was observed(Giovanni et al., 2010). This is in comparison to a 57% fat gain noted in wild-type littermates. Experiments on germ-free FIAF KO mice fed with a high fat, containing high-carbohydrate levels were not protected from diet-induced obesity. Consequently, the blunted FIAF expression might have led to the accumulation of triglyceride in adipocytes and adipose tissue hypertrophy of conventionalized germ-free mice (Giovanni et al., 2010).

It is well-established that environmental factors including host diet influence the relative representation of certain bacterial phyla in the gut. Beyond population structure, it is clear that functional attributes of a community may drastically influence host metabolism (Martin and Sela, 2013). Nutrition influences the composition of the gut microbiome such as high fiber food that passes to the colon (Khan et al., 2014; Song et al., 2016). For this reason, studies showed more diversified levels of GM in rats reared on high fiber diets (Blaut and Klaus, 2012). Fermentable fibers are utilized by specific microbiota capable of metabolizing the molecular structure of a given substrate. As an example, dietary oligosaccharides including plant and milk-derived stimulate the growth of Bifidobacteria (LoCascio et al., 2007; Meyer and Stasse-Wolthuis, 2009). Mouse studies confirm this, as the GM shifts when standard chow is changed to a semi-synthetic high-fat diet due to a lower percentage of fermentable dietary fibers (Fleissner et al., 2010).

There are several disparities regarding the relative ratio of Firmicutes/Bacteroidetes in obese individuals compared to healthy subjects (Santacruz et al., 2010). Interestingly studies conducted in developed countries, higher ratios of Firmicutes to Bacteroidetes have been linked to obesity. In a comparative study, Bacteroidetes dominated the microbiota of children from rural African villages, while Firmicutes dominated the microbiota of European children (De Filippo et al., 2010). Differential GM composition was attributed to diet as well as ethnicity, sanitation, hygiene, geography, and/or climate (De Filippo et al., 2010). Bacteroidetes dominated the microbiota of children from rural African villages while Firmicutes dominated the microbiota of European children. Differential GM composition was attributed to diet as well as ethnicity, sanitation, hygiene, geography, and/or climate (De Filippo et al., 2010). Other studies did not identify such a correlation and reported that the relative abundance of Firmicutes decreased following gastric bypass surgery (De Filippo et al., 2010). Moreover, obese pregnant women exhibited a decrease in *Bifidobacterium* sp. and Bacteroidetes with an increase in Prevotellaceae (Santacruz et al., 2010).

There have been attempts to define a “core microbiome” as some have speculated that deviation from a core structure may lead to pathologies such as obesity (Turnbaugh et al., 2009; Kovatcheva-Datchary and Arora, 2013). Although a conserved core microbiome structure is difficult to identify due to high inter-individual variation (Qin et al., 2010), community function may be conserved within this variation. It is noteworthy that GM of obese individuals exhibit less taxonomic richness and potentially diminished metabolic capacity than the microbiota of lean individuals (Turnbaugh et al., 2009; Greenblum et al., 2012; Kovatcheva-Datchary and Arora, 2013).

In studies of obese and lean twins, microbial groups were found correspondingly enriched or depleted in the lean and obese subjects (Turnbaugh et al., 2009). Most of the microbial genes that were linked with obesogenic pathways did not belong to Bacteroidetes but came from either Actinobacteria (75%) or Firmicutes (25%). In a follow-up study conducted on mice, the microbiome composition shifts in just 1 day after switching mice from low fat (rich in fiber) to a high-fat diet with increased adiposity occurring just 2 weeks (Turnbaugh et al., 2009). Bacteroidetes were enriched in the GM in mice consuming a high fiber diet, as they are efficient utilizers of plant polysaccharides (Maslowski and Mackay, 2011). This is in contrast to the Firmicute enrichment and associated obesogenic microbiota in high caloric diets typically encountered in developed countries. Future research should focus on assessing the possibility of altering the Firmicutes to Bacteroidetes ratios as a therapeutic solution to obesity. Such an alteration may be achieved by the use of either antimicrobial agents (Morgun et al., 2015) and/or the use of probiotics in conjunction with changing the selective environment induced by switching to a low-fat/high-fiber diet.

Aspects related to understanding the relationship between GM in relation to Obesity:

There are still lots of pitfalls regarding the understanding of the relationship between diet, lifestyle activities, GM, and obesity. Most of the investigations concentrated on GM in the gastrointestinal tract. However, other important aspects have been overlooked and include the following (Conlon and Bird, 2014):

1. Better understanding of the functions of GM and their physiological relevance to obesity.
2. Identification of the different types of genes of the current GM and the many more newly discovered ones and their function in breaking down of the different types of foods, their role in energy harvest and the resulting by-product(s)in relation to obesity.
3. Focus should also be made on understanding the role of bacteria in the small intestine (ileum and cecum) and the role of diet in the overall health of the small intestine, which may become inflamed because of imbalances in gut microbial populations in addition to delineating the involvement of bacteria in the small intestine enteropathies, leaky gut, and obesity.
4. Understanding the influence of the different types of foods that have the ability to compromise the integrity of the gut mucosa, which is considered of critical importance to the overall health and to obesity. Diets should be tailored toward foods that keep the gut mucosa intact and prevent invading microbes from causing inflammation in the gut mucosa.
5. Delineating the association between GM and immune system; such an understanding will reveal how GM can contribute to obesity or not and such an information can be used to design diets to promote optimal body weight.
6. Understanding the bidirectional communication of data between GM on the satiety center in the brain and their effect on obesity.
7. Effect of dietary manipulation affects GM composition among individuals and their effects on mode swings and food intake and their consequences on obesity.
8. Food by-products effect on GM and in turn on obesity.
9. Understanding the inter-individual variations of GM and their role in obesity.
10. The modulation of the microbial profile based on the understanding the origins and variations to fight obesity.
11. Understanding the genetic predisposition to obesity and how environmental factors and diet can be modified to overcome this problem. This may result in the development of diets with an optimum composition to enhance the growth of certain bacteria that will counteract the effect of the genetic factors of the host that can better resist obesity.
12. The effect of long-term dietary habits on the microbial populations in the GM.
13. Effect of long-term administration of vitamins, amino acids, and micronutrients including phytobiologics on the GM composition and their consequences on obesity.
14. Understand how the main constituents of the diet affect GM composition and their effect on obesity.
15. The effect of food matrix, structure, the particle size, and the interaction of the food matrix with the gastric enzymes found in the gut.
16. The influence of cooking food or consuming it raw and its effects on GM and its relation to obesity.
17. The effects of prebiotics and probiotics on the GM and their relationship to obesity.

The role of GM may contribute to the increased incidence of autoimmune and metabolic diseases in developed countries. An example is Type-1 and type-2 diabetes. Understanding the role that GM plays in these two diseases may result in prevention and intervention strategies to control those two diseases. However, well-controlled prospective human studies are required for the understanding of the role of the GM and its response to environmental factors in order to identify effective preventive strategies targeting specific component of the gut ecosystem (Nobel et al., 2015).

## Conclusion

During the last decade or two, there has been an exponential increase due to “omics” technologies in our understanding of the composition and functions of the human GM. The “omics” technologies have made it easier to analyzed on a large large-scale of the genetic and metabolic profile of microbial communities, thus offering the possibility of a new route for therapeutic intervention. Their association is similar to what has been seen in the immune system, which comprises a collection of cells that work in harmony either with the host in order to promote health or in other instances cause disease. Obesity is a major problem worldwide and any possible was should be used to control it. Scientists worldwide are trying to use different and latest technologies to treat obesity. It well-documented that there are different factors that contribute to obesity other than the increased energy harvest from the diet. It is extremely necessary to decipher the underlying association between the adipose tissue and pathophysiology in relation to different metabolic disorders including but not only limited to obesity (DiBaise et al., 2012; Pekkala et al., 2015).

As such, different mechanisms have been proposed to link to the composition. This field is still is in its early stages and require elaborately controlled studies to substantiate the available data that link obesity to GM. Based on the above, it is evident that better understanding of the role of GM in obesity would lead to possible solutions to manage this disorder. The GM compositions are genetically predisposed and are affected by the type of diet consumed and its role in the treatment of obesity should not be overlooked. Future work should be conducted in a very controlled manner to have a reproducible data that will delineate the role of microbiota in obesity across the globe. For this reason, there is an urgent need for an open and enhanced dialogue between experimental scientists and computational scientists to uncover possible reasons behind the conflicting results that are published and to: get novel findings in the field, synthesize diverse information related to GM and obesity and accelerate the future of discovering new therapeutic applications.

Finally, it would be of interest to identify different bacterial phyla that are involved with obesity and treatment of obesity can be achieved via the modification of the microbiota of the obese persons. Such a process may be achieved by transplantation of known microbiota that are not involved in obesity.

## Author contributions

SH and IK contributed to main content of design and drafting of this article. EB and TK helped in critically reviewing the article. SBA, SB, SMA, GA, and EA contributed to literature survey and writing.

### Conflict of interest statement

The authors declare that the research was conducted in the absence of any commercial or financial relationships that could be construed as a potential conflict of interest.
